# Biological Activities and Phytochemical Profile of Hawm Gra Dang Ngah Rice: Water and Ethanolic Extracts

**DOI:** 10.3390/foods14071119

**Published:** 2025-03-24

**Authors:** Suchanat Chaithong, Pinwadee Sukkarn, Chakkapat Aenglong, Wanwipha Woonnoi, Wanwimol Klaypradit, Wiwit Suttithumsatid, Narainrit Chinfak, Jirawat Seatan, Supita Tanasawet, Wanida Sukketsiri

**Affiliations:** 1 Division of Health and Applied Sciences, Faculty of Science, Prince of Songkla University, Hat Yai, Songkhla 90110, Thailand; 2 Division of Physical Science, Faculty of Science, Prince of Songkla University, Hat Yai, Songkhla 90110, Thailand; 3 Department of Agro-Industrial, Food and Environmental Technology (AFET), Faculty of Applied Science, King Mongkut’s University of Technology North Bangkok (KMUTNB), Bangsue, Bangkok 10800, Thailand; 4 Department of Fishery Products, Faculty of Fisheries, Kasetsart University, Bangkok 10900, Thailand; 5 Department of Pharmacognosy and Pharmaceutical Botany, Faculty of Pharmaceutical Sciences, Prince of Songkla University, Hat Yai, Songkhla 90110, Thailand; 6 Phytomedicine and Pharmaceutical Biotechnology Excellence Center, Faculty of Pharmaceutical Sciences, Prince of Songkla University, Hat Yai, Songkhla 90110, Thailand; 7 Department of Marine Science, Faculty of Science, Chulalongkorn University, Bangkok 10330, Thailand

**Keywords:** antioxidant activity, antiproliferative, lung cancer, breast cancer, colored rice

## Abstract

Hawm Gra Dang Ngah rice (HDNR) is a red rice variety cultivated in Thailand’s southern border region, yet its biological properties have not been extensively studied. This study investigates the effects of HDNR extracts on bioactive constituents, spectral fingerprints, and antioxidant capacities. We evaluated the inhibitory effects of aqueous (HDNR-W) and ethanolic (HDNR-E) extracts on monoamine oxidase (MAO), α-glucosidase, and HMG-CoA reductase activities, as well as their cytotoxicity in normal and cancer cells. The results demonstrated that HDNR-E contained significantly higher concentrations of phenolic compounds, flavonoids, and anthocyanins compared to HDNR-W. In contrast, HDNR-W exhibited greater amino acid content than HDNR-E. FT-IR analysis revealed solvent-specific interactions that influenced compound solubility, highlighting distinct extraction efficiencies. Antioxidant assays showed HDNR-E to be markedly more potent, with superior performance in DPPH, ABTS, metal chelation, and FRAP assays, as evidenced by its lower IC_50_ values relative to HDNR-W. Furthermore, HDNR-E displayed significantly stronger inhibitory activity against both MAO and α-glucosidase compared to HDNR-W. Conversely, HDNR-W demonstrated greater inhibitory efficacy toward HMG-CoA reductase than HDNR-E. Furthermore, HDNR-E exhibited significant antiproliferative effects against A549 lung cancer and MCF-7 breast cancer cells without affecting normal cells. These results highlight the potential of HDNR-E as a valuable source of bioactive compounds and underscore the importance of solvent selection in enhancing the health benefits of rice extracts.

## 1. Introduction

Non-communicable diseases (NCDs) constitute a significant global health challenge, characterized by their chronic nature and the absence of transmissibility between individuals. These conditions arise from a complex interplay of genetic factors, physiological traits, environmental conditions, and lifestyle habits. NCDs pose a significant global health challenge, accounting for approximately 74% of all deaths worldwide. The leading causes of NCDs include cardiovascular diseases, cancers, chronic respiratory diseases, and diabetes, which collectively are responsible for over 80% of premature NCD-related deaths [1,2]. Research highlights the importance of medicinal plants in rural communities, such as those in Thailand, where local populations utilize a variety of plant species to manage health issues like diabetes, hypertension, and chronic respiratory ailments [3]. The investigation of natural products holds considerable potential for addressing the challenges posed by NCDs. This approach merges traditional knowledge with contemporary scientific research to formulate effective therapeutic strategies.

Rice (*Oryza sativa* L.) is one of the world’s three main staple foods, serving as a primary source of nutrition for over half of the global population [4]. This grain is rich in proteins (comprising 6–7% of total content), with specific types including 10-prolamins, 13-prolamins, and 16-prolamins. It is also abundant in minerals, vitamins, dietary fibers, unsaturated fatty acids, polysaccharides, and polyphenols. These bioactive compounds are primarily located in the pericarp, seed coat, aleurone layer, germ, and endosperm [5,6]. Health-conscious consumers are increasingly choosing colored rice varieties for their superior nutritional profiles. Research indicates a strong link between rice color and flavonoid content, with genetic and environmental factors influencing the phytochemical differences among pigmented rice grains [7]. In Thailand, various pigmented rice cultivars, such as Sanyod rice and Riceberry rice, are gaining recognition for their health-promoting properties. These rice varieties are rich in antioxidants, phytochemicals, and other beneficial compounds that offer numerous health benefits [8,9]. Hawm Gra Dang Ngah rice is a variety of red rice grown in the Takbai district of Narathiwat, which is situated in the southern border region of Thailand. However, the current production levels of this rice are relatively low [10]. While numerous studies have examined the biological properties of color pigments in other rice varieties, such as Sangyod red rice [8,9,11], Kum Doi Saket [12], and Dawk Mali 105 [13], there remains a notable gap in focused research on the biological properties and bioactive components specifically present in Hawm Gra Dang Ngah rice. This study aims to conduct a comparative analysis of extracts from Hawm Gra Dang Ngah rice, utilizing both ethanol and water as solvents. The primary objective is to evaluate how solvent selection influences the levels of key compounds, including phenolics, flavonoids, and anthocyanins, which are important for their health benefits and for enhancing the sensory qualities of food products containing these rice extracts. Additionally, the study also explores the inhibition of monoamine oxidase (MAO), α-glucosidase, and HMG-CoA reductase activities, as well as the cytotoxic effects of these extracts on both normal and cancer cells.

## 2. Methods and Materials

### 2.1. Materials and Chemical Reagents

Hawm Gra Dang Ngah rice was sourced from Narathiwat, Thailand. Absolute ethanol was purchased from the Liquor Distillery Organization in Thailand. The reagents 2,2-diphenyl-1-picrylhydrazyl (DPPH; CAS No. 1898-66-4), 2,2′-azino-bis(3-ethylbenzthiazoline-6-sulphonic acid) (ABTS; CAS No. 30931-67-0), 2,4,6-tri(2-pyridyl)-s-triazine (TPTZ; CAS No. 3682-35-7), and ferrozine (CAS No. 63451-29-6) were obtained from Sigma-Aldrich (St. Louis, MO, USA). Additionally, potassium peroxodisulfate (K_2_S_2_O_8_; CAS No. 7727-21-1), iron(III) chloride hexahydrate (FeCl_3_·6H_2_O; CAS No. 10025-77-1), and iron(II) sulfate heptahydrate (FeSO_4_·7H_2_O; CAS No. 7782-63-0) were supplied by Merck (Darmstadt, Germany). All other chemicals and reagents used were of analytical reagent grade and readily available commercially.

### 2.2. Extraction of Hawm Gra Dang Ngah Rice

Hawm Gra Dang Ngah rice underwent a precise grinding process using a blender. The rice extraction method was modified from our previously described protocols [9,11]. The resulting fine powder was then passed through a laboratory test sieve with a mesh size of 4.75 mm (4 Mesh). This rice powder was extracted using two different solvents. For the first extraction, rice powder was combined with deionized water at a ratio of 1:5 and heated to 50 °C for 1 h. After that, the mixture was centrifuged at 7000× *g* for 15 min and lyophilized using a freeze dryer (LaboGene, Allerød Municipality, Denmark) to yield the Hawm Gra Dang Ngah rice water extract (HDNR-W). In the second extraction, another portion of the rice powder was mixed with 70% ethanol in a 1:10 ratio and allowed to extract for 72 h, followed by centrifugation at 7000× *g* for 15 min, and the solvent was evaporated using a rotary evaporator (Rotavapor R-220 Pro, Flawil, Switzerland) to produce the Hawm Gra Dang Ngah rice ethanolic extract (HDNR-E). The lyophilized powders from both extracts were stored in amber glass bottles and placed in a desiccator at −20 °C for preservation until further use.

### 2.3. Determine the Bioactive Compound Content in HDNR-W and HDNR-E

The total phenolic content was evaluated using the Folin–Ciocalteu colorimetric method, with slight modifications based on the procedure described by Aenglong et al. [8]. Briefly, 25 μL aliquots of extracts (10 mg/mL) or standard solutions were mixed with 50 μL deionized water and 50 μL 10% (*v*/*v*) Folin-Ciocalteu reagent. The reaction mixture was neutralized with 100 μL of 7.5% (*w*/*v*) sodium carbonate solution. After incubation for 1 h at room temperature, absorbance was measured at 765 nm using a microplate reader. A calibration curve was generated using gallic acid standard solutions. The results were expressed as micrograms of gallic acid equivalent per milligram of the sample, calculated on a dry weight basis.

The total flavonoid content was determined using a colorimetric method with minor modifications adapted from Aenglong et al. [8]. Specifically, 20 μL aliquots of appropriately diluted extracts or standard solutions were mixed with 120 μL of deionized water and 10 μL of 5% (*w*/*v*) NaNO_2_. After 5 min, 10 μL of 10% (*w*/*v*) AlCl_3_·6H_2_O solution was added, and the mixture was incubated for an additional 5 min. Subsequently, 50 μL of 1 M NaOH was introduced. Following thorough mixing, the reaction solution was allowed to stand undisturbed for 15 min before measuring absorbance at 510 nm using a microplate reader. The total flavonoid content was reported as micrograms of quercetin equivalent per milligram of dry weight.

The total anthocyanin content was assessed using the pH differential method as described by Lee et al. [14]. The HDNR-W and HDNR-E extracts were diluted separately with a solution containing 0.4 M sodium acetate buffer (pH 4) and 0.025 M potassium chloride (pH 1). Absorbance values were measured at 700 nm and 510 nm using a microplate reader. The concentration of anthocyanin pigments, expressed as cyanidin-3-glucoside (CG) equivalents, was calculated using a specific Equation (1).

(1) $$\mathrm{CG}\;\mathrm{equivalents}\;(\mathrm{mg}/L)=\frac{A\;\times \;MW\;\times \;\mathrm{DF}\;\times \;1000}{ɛ\;\times \;1}$$

where A = (A_520nm_ − A _700nm_)_pH 1.0_ − (A_520nm_ − A_700nm_)_pH 4.5_

MW = molecular weight of CG is 449.2 g/mol

DF = dilution factor

l = pathlength in cm

ɛ = molar extinction coefficient for CG is 26,900 L mol^−1^ cm^−1^

1000 = factor for conversion from g to mg

### 2.4. UHPLC-ESI-Q-TOF-MS/MS

The phytochemical composition of HDNR-W and HDNR-E was determined using liquid chromatography coupled with tandem mass spectrometry (LC-MS/MS), with modifications based on Woonnoi et al. [11] and Hanchang et al. [9]. The analysis utilized a Zorbax Eclipse Plus C18 reverse phase column, measuring 150 × 2.1 mm with a particle size of 1.8 µm. The HDNR-W and HDNR-E samples were prepared in a solution containing 0.1% formic acid, achieving a final concentration of 10 mg/mL. Prior to injection, the solutions were filtered through a 0.22 µm syringe filter to ensure clarity and prevent contamination. The injection was performed at a flow rate of 0.2 mL/min over a total duration of 28 min. The LC-MS/MS analysis was conducted using an Ultra-High-Performance Liquid Chromatography (UHPLC) system from Agilent Technologies (Santa Clara, CA, USA), equipped with an Electrospray Ionization-Quadrupole-Time of Flight Mass Spectrometer. The elution gradients included eluent A (0.1% formic acid in water) and eluent B (acetonitrile), following a specific multistep linear progression. The column temperature was maintained at 30 °C throughout the analysis, with a 2 µL injection volume for both positive and negative ionization modes. Data analysis was performed using MassHunter WorkStation and METLIN Metabolite Software version 8 for accurate spectral identification and comparison against a comprehensive library.

### 2.5. Amino Acid Profile

The method for analyzing amino acids in the rice extract was modified from our previously described protocols [8]. The extract samples were hydrolyzed in 6 N HCl under vacuum at 110 °C for 24 h. After hydrolysis, the dried residues were homogenized in 0.02 N HCl. The resulting solution was then filtered through a 0.22 µm filter. Finally, 20 µL of the filtered sample was injected into the amino acid analyzer (Hitachi L-8080, Tokyo, Japan) for analysis.

### 2.6. Fatty Acid Profile

The fatty acid profile was analyzed using the methodology described by Curtis et al. [15]. Samples weighing 1 g were extracted with a mixture of chloroform, methanol, and distilled water in a 1:1:1 ratio. The extraction process involved shaking the samples at 150 rpm for 15 min, followed by centrifugation at 10,000 rpm for 15 min to separate the chloroform phase. The chloroform phase, which contained the fatty acids, was then evaporated at 50 °C using parallel evaporators (Multivapor™, Buchi, Flawil, Switzerland). The resulting oil was diluted with 1 mL of isooctane and transferred to a test tube for conversion into fatty acid methyl esters (FAME). A 1.0 μL aliquot of the FAME sample was analyzed using a gas chromatography system (7890A, Agilent, Santa Clara, CA, USA) equipped with an HP-88 capillary column (100 m × 0.25 mm, 0.20 µm) for separation. Fatty acids were identified by comparing the sample peaks to those of a chromatographic standard mixture (Sigma 47,885-U Supelco 37 Component FAME Mix; Bellefonte, PA, USA). The results were expressed as a percentage of the total fatty acid composition in the sample.

### 2.7. Heavy Metal and Trace Element in HDNR-W Extracts

The method for analyzing heavy metals and trace elements in the rice extract was modified from the protocol described by Sneddon et al. [16]. Briefly, 1 g of wet sample was placed in crucibles and ashed at 550 °C for 4 h in a muffle furnace. Any remaining carbonates were removed by treating the ash with 6 M HCl. The solution was then evaporated almost to dryness on a hot plate, and the residue was dissolved in 0.1 M HNO_3_. The clear solution was transferred into a polyethylene bottle and diluted to 20 mL with 0.1 M HNO_3_. Subsequently, the solution was evaporated to dryness, reconstituted with 30 mL of deionized water, and filtered through a 0.45 μm syringe filter. A Perkin Elmer OPTIMA 2000 DV model (Waltham, MA, USA) of an Inductively Coupled Plasma-Optical Emission Spectrometer (ICP-OES) was used to measure the heavy metal concentrations.

### 2.8. Fourier Transform Infrared Spectroscopy (FT-IR) Analysis

The FT-IR spectra of the HDNR-W and HDNR-E extracts were analyzed using an FT-IR spectrometer (Invenio S, Bruker, Billerica, MA, USA) at room temperature. The measurements covered a range from 4000 to 400 cm^−1^ within the mid-infrared region. Signal acquisition was performed in automatic mode, and the resulting spectral data were analyzed using the OPUS 3.0 software (Bruker).

### 2.9. Antioxidant Assay

The antioxidant properties of the HDNR-W and HDNR-E extracts were assessed using a series of assays, including the DPPH assay, ABTS assay, metal chelating activity (MCA), and ferric reducing antioxidant power (FRAP) assay.

The scavenging activity of the DPPH radical was assessed using the method previously described by Aenglong et al. [8]. The effectiveness of the test solution in scavenging the DPPH radical was quantified using a calibration curve based on ascorbic acid. The results were expressed as micrograms of sample equivalent to ascorbic acid.

For the ABTS assay, the radical scavenging capacity of the HDNR-W and HDNR-E extracts was determined using the method outlined by Aenglong et al. [8]. The ABTS radical scavenging was calculated using a calibration curve based on ascorbic acid, and the results were expressed as micrograms of sample equivalent to ascorbic acid.

The MCA was assessed following the method outlined by Chotphruethipong et al. [17]. The reaction mixture consisted of 10 μL of 2 mM FeCl_2_, 20 μL of 5 mM ferrozine, and each extract, which was incubated at room temperature for 20 min. The absorbance was then measured at 562 nm using a microplate reader. The activity was expressed as micrograms of EDTA equivalents.

The FRAP assay was conducted at a wavelength of 593 nm, following the method previously described by Aenglong et al. [8]. Both HDNR-W and HDNR-E extracts were combined with the FRAP reagent and incubated at room temperature for 30 min. The FRAP value for each extract was determined using a calibration curve based on ferrous sulfate standards.

### 2.10. Inhibition of HMG-CoA Reductase Activity of HDNR-W and HDNR-E

The method for evaluating the inhibitory activity of HMG-CoA reductase was modified from our previously described protocols [11]. The inhibitory potential of HMG-CoA reductase in the HDNR-W and HDNR-E extracts was assessed using the HMG-CoA reductase assay kit (Sigma-Aldrich Co., St. Louis, MO, USA), according to the manufacturer’s instructions at a wavelength of 340 nm. Pravastatin served as a positive control drug. The degree of HMG-CoA reductase inhibition was calculated using the following equation:

$$\mathrm{Inhibition}\;\mathrm{activity}\;(\%)=\frac{{\mathrm{Abs}}_{\mathrm{control}\;}-{\mathrm{Abs}}_{\mathrm{test}}}{{\mathrm{Abs}}_{\mathrm{control}}}\;\times 100$$

### 2.11. Inhibition of α-Glucosidase Activity of HDNR-W and HDNR-E

The inhibitory activity of α-glucosidase was evaluated according to the method outlined by Suttithumsatid et al. [18], with slight modification. HDNR-W and HDNR-E were dissolved in DMSO, and 20 μL of each sample was mixed with an equal volume of a 0.1 Unit/mL α-glucosidase enzyme solution in a 96-well plate. The mixture was then incubated at 37 °C for 10 min. Subsequently, p-nitrophenyl-α-d-glucopyranoside (pNPG) was added, and the mixture was incubated again at 37 °C for 40 min. To stop the reaction, 80 μL of 0.2 mM sodium carbonate in phosphate buffer was added to each well. The resulting concentration of p-nitrophenol was measured using a microplate reader at 405 nm. A blank control was created by replacing the α-glucosidase solution with a boiled enzyme solution, while a control experiment utilized DMSO and deionized water instead of the sample solution. All experiments were performed in triplicate. The percentage inhibition was calculated using the following formula:

%Inhibition α-glucosidase activity = (A_c_ − A_b_) − (A_s_ − A_b_)/(A_c_ − A_b_) × 100

where A_c_ is the absorbance of the control, A_s_ is the absorbance of the sample, and A_b_ is the absorbance of the blank.

### 2.12. Inhibition of Monoamine Oxidases (MAO) Activity of HDNR-W and HDNR-E

The inhibitory activity of MAO was evaluated using a method adapted from Boonruamkaew et al. [19], with minor modifications. An MAO activity assay was conducted using the MAO inhibitor screening kit (Sigma Aldrich, St. Louis, MO, USA). MAO-A and MAO-B were prepared, and the test samples (HDNR-W and HDNR-E) were dissolved in DMSO. The enzyme and test samples were added to a 96-well microplate and incubated at room temperature for 10 min. Subsequently, benzylamine and a chromogenic solution (comprising 5 mM vanillic acid, 2.5 mM 4-aminoantipyrine, and 4 U/mL horseradish peroxidase type II) were introduced. This combination initiated the reaction, which proceeded for 1 h at room temperature before detection at 490 nm.

### 2.13. Cell Culture and Cytotoxicity of HDNR-W and HDNR-E in Normal and Cancer Cell Lines

MRC-5 (CCL-171™), hTERT-HME1 (CRL-4010™), A549 (CCL-185™), and MCF-7 (HTB-22™) cell lines were obtained from the American Type Culture Collection (ATCC). MRC-5, hTERT-HME1, and A549 cells were cultured in Dulbecco’s Modified Eagle Medium, while MCF-7 cells were maintained in the Minimum Essential Medium Eagle alpha medium (Gibco, Waltham, MA, USA) [20]. Both media were supplemented with 10% fetal bovine serum (Gibco, USA), 1% penicillin-streptomycin (Gibco, USA), and 1% L-glutamine (Gibco, USA). Once the cells reached 80–90% confluence, they were seeded into 96-well plates at a density of 10^4^ cells per well. Subsequently, HDNR-W and HDNR-E at concentrations of 0, 0.5, 1, 5, 10, 25, 50, 100, 250, and 500 μg/mL were added and incubated for 24 h. The cytotoxicity of both extracts was evaluated using the 3-(4,5-dimethylthiazol-2-yl)-2,5-diphenyltetrazolium bromide (MTT) assay at a wavelength of 570 nm with a microplate reader.

### 2.14. Statistical Analysis

All data were expressed as mean values ± standard deviation. The chemical and biochemical analyses were assessed using the *t*-test for pairwise comparisons. In the cell culture model, one-way ANOVA was performed to detect variances among multiple groups, followed by the Duncan post hoc test. Statistical significance was established at *p* ≤ 0.05.

## 3. Results

### 3.1. Major Components of HDNR-W and HDNR-E

**Figure 1:**
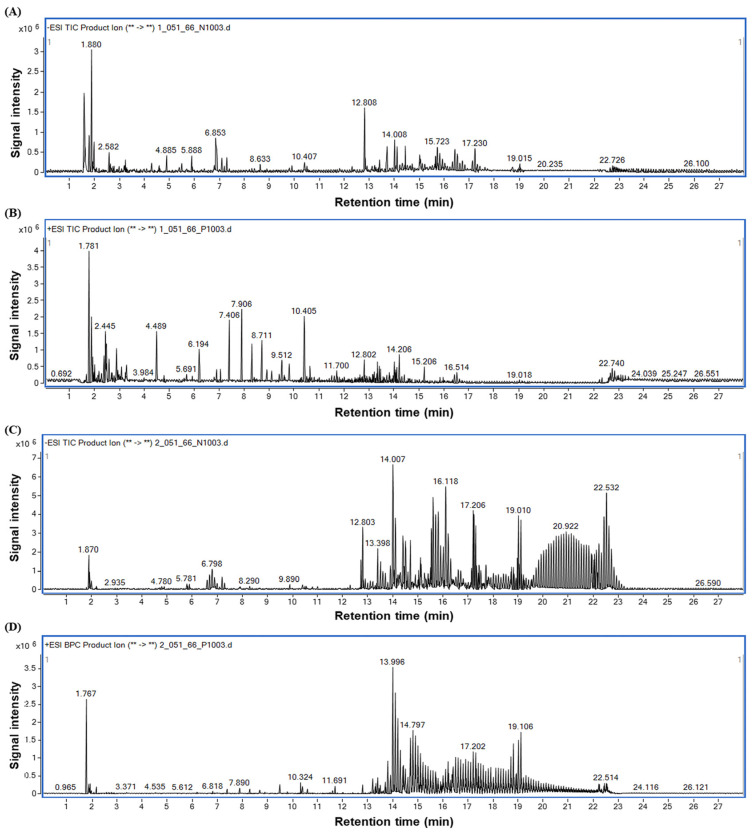
LC-MS/MS analysis of HDNR-W in negative (**A**) and positive (**B**) modes and LC-MS/MS analysis of HDNR-E in negative (**C**) and positive (**D**) modes. The asterisk (*) has no meaning.

The identification of compounds in HDNR-W and HDNR-E was performed using UHPLC-QTOF-MS analysis. The peak chromatograms of HDNR-W were shown in Figure 1A,B, displaying data in both negative and positive modes, respectively. Similarly, Figure 1C,D illustrates the peak chromatograms of HDNR-E in both modes.

The compounds detected in HDNR-W and HDNR-E were summarized in Table 1 and Table 2. The UHPLC-QTOF-MS analysis revealed a diverse array of compounds, including phenolics, flavonoids, alkaloids, and glycosides. Additionally, both HDNR-W and HDNR-E contained polysaccharides, fatty acids, and amino acids. These results highlight the presence of a wide range of biological compounds in HDNR-W and HDNR-E.

### 3.2. Amino Acid Profile in the HDNR-W and HDNR-E Extracts

The composition of essential amino acids (EAAs) and non-essential amino acids (NEAAs) in the HDNR-W extract was significantly higher than that in the HDNR-E extract, as demonstrated in Table 3. The concentrations of histidine (His; 0.48 mg/100 g), isoleucine (IIe; 0.41 mg/100 g), lysine (Lys; 0.93 mg/100 g), methionine (Met; 0.29 mg/100 g), threonine (Thr; 1.13 mg/100 g), and valine (Val; 1.22 mg/100 g) were notably greater in the HDNR-W extract compared to the HDNR-E extract. Conversely, leucine (Leu; 0.76 mg/100 g) and phenylalanine (Phe; 0.50 mg/100 g) exhibited the opposite trend, with slightly higher concentrations in the HDNR-E extract than in the HDNR-W extract. Furthermore, NEAAs showed significant differences, with higher concentrations observed in the HDNR-W extract compared to the HDNR-E extract. There were also notable variations in the concentrations of hydrophobic amino acids (HPBs) and hydrophilic amino acids (HPLs) between the two extracts, underscoring the impact of extraction solvents on amino acid yield. Regarding non-protein amino acids (NPAAs), the HDNR-W extract exhibited higher levels of β-alanine and γ-aminobutyric acid (GABA), while the HDNR-E extract contained greater amounts of taurine (Table 3).

### 3.3. Fatty Acid Profile in the HDNR-W and HDNR-E Extracts

The examination of fatty acid composition revealed clear differences between HDNR-W and HDNR-E, as shown in Table 4. A significant variation between the two samples was evident in the types of fatty acids identified. These findings demonstrated that although both extracts contain some common fatty acids, including C14:0, C16:0, and C18:0, HDNR-E displays a wider variety of detected fatty acids than HDNR-W. Several fatty acids were not detected (ND) in HDNR-W but were present in HDNR-E. Notably, HDNR-W did not show the presence of fatty acids such as C20:0, C21:0, C22:0, and C24:0, all of which were presented in HDNR-E. Furthermore, HDNR-E also contains detectable levels of C18:3n3 and C20:2, which were absent in HDNR-W. This variation in fatty acid detection indicates that the ethanol extraction method used for HDNR-E may have facilitated the extraction of a wider range of lipid compounds compared to the water extraction method applied to HDNR-W.

### 3.4. Heavy Metal Concentration in HDNR-W

Table 5 demonstrates the concentrations of heavy metals in HDNR-W. The levels of arsenic (As), cadmium (Cd), chromium (Cr), copper (Cu), nickel (Ni), and lead (Pb) were found to be below the maximum allowable limits established by the Thai FDA [21]. Furthermore, the essential elements manganese (Mn), zinc (Zn), and iron (Fe) were detected in the following descending order: Mn > Zn > Fe.

### 3.5. Total Phenolic, Total Flavonoid, and Total Anthocyanin Contents in the HDNR-W and HDNR-E Extracts

**Figure 2:**
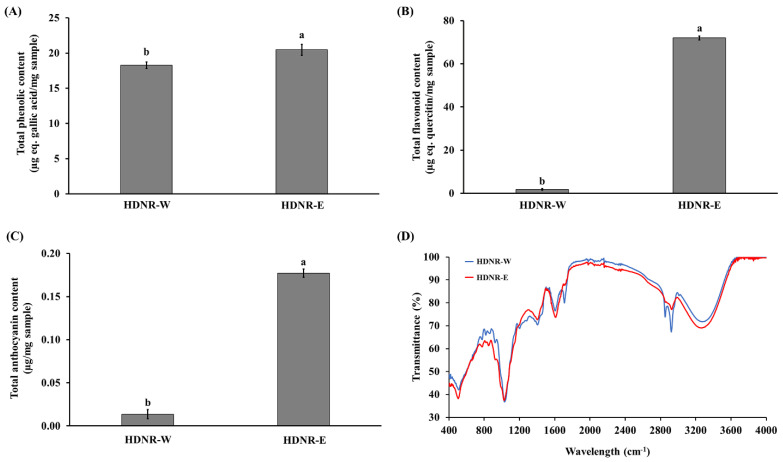
Total phenolic content (**A**), total flavonoid content (**B**), total anthocyanin content (**C**), and the FT-IR spectra (**D**) of HDNR-W and HDNR-E. Data are presented as mean ± standard deviation (*n* = 3). The lowercase letters indicate statistical significance (*p* ≤ 0.05).

As shown in Figure 2A–C, the total phenolic, total flavonoid, and total anthocyanin contents in the HDNR-E extract were significantly higher (*p* ≤ 0.05) than those in the HDNR-W extract. The concentrations of total phenolic, flavonoid, and anthocyanin in the HDNR-E extract were 20.47 ± 3.71, 72.02 ± 3.72, and 0.18 ± 0.01 μg/mg of sample, respectively. On the other hand, the HDNR-W extract contained total phenolic, flavonoid, and anthocyanin contents of 18.27 ± 2.99, 1.77 ± 0.27, and 0.014 ± 0.00 μg/mg of sample, respectively.

### 3.6. FT-IR in the HDNR-W and HDNR-E Extracts

As illustrated in Figure 2D, the FTIR spectra of HDNR-W and HDNR-E exhibited notable similarities, with distinct band intensities reflecting the presence of identical functional groups at varying concentrations. The differences in band intensity and pattern between the two extracts reflect the unique solubility and interactions of the compounds with their respective solvents. In the carbonyl (C=O) stretching region (1700–1750 cm^−1^), both samples displayed bands indicative of carbonyl-containing compounds, including fatty acids and amines. The hydroxyl group (-OH) region (3200–3600 cm^−1^) showed a broad band in HDNR-W, suggesting the presence of hydroxyl-containing compounds, while HDNR-E exhibited similar bands with varying intensities due to ethanol’s specific interactions. Distinct spectral features in the 3000–2800 cm^−1^ range, attributed to C-H stretching vibrations from lipid backbones, indicated differences in lipid extraction efficiency between the solvents. Bands in the aromatic region suggest the presence of phenols and flavonoids, with variations in intensity reflecting discrepancies in extraction efficacy. The carbohydrate region (1000–1100 cm^−1^) reveals bands associated with carbohydrate components, indicating that HDNR-W may possess a unique carbohydrate profile. In the amide (1650–1550 cm^−1^) and amine (3400–3100 cm^−1^) regions, bands signified proteinaceous compounds and amino acids. Although both solvents showed similar bands, variations in intensity suggest differences in extraction efficiency. Additionally, bands at 1318 cm^−1^ and 1052 cm^−1^ corresponded to C-O stretching in cellulose/hemicellulose, while a band at 1150 cm^−1^ indicates C-O-C asymmetric bridge stretching. The minor band at 898 cm^−1^ signifies the β-1,4 glycosidic linkage within cellulose. Overall, the observed differences in band intensities and patterns across the FTIR spectra of HDNR-W and HDNR-E can be attributed to the varying solubility, polarity, and interactions of compounds with each solvent.

### 3.7. Antioxidation Activity of HDNR-W and HDNR-E Extracts

**Figure 3:**
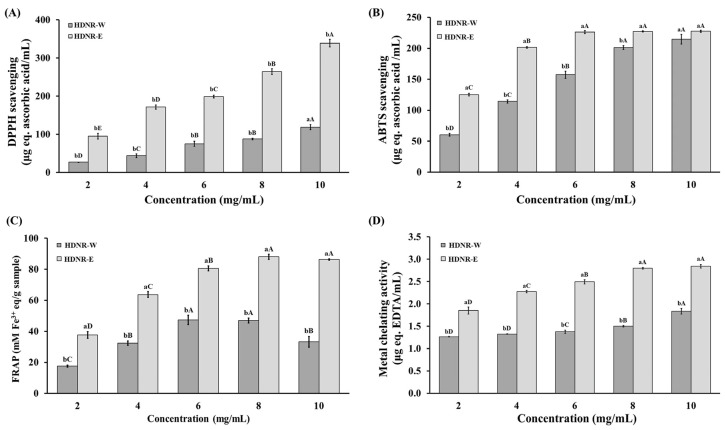
The antioxidation activity of HDNR-W and HDNR-E. (**A**) DPPH scavenging, (**B**) ABTS scavenging, (**C**) ferric ion reducing antioxidant power (FRAP), and (**D**) metal chelating activity (MCA). Data are expressed as mean ± standard deviation (*n* = 3). Lowercase letters are used to indicate statistical significance between HDNR-W and HDNR-E (*p* ≤ 0.05), while uppercase letters are used to denote significant differences observed at various concentrations within the same extract (*p* ≤ 0.05).

The DPPH radical (DPPH·) scavenging capacity of HDNR-W and HDNR-E was demonstrated in Figure 3A. Both extracts exhibited concentration-dependent scavenging activity, with HDNR-E showing markedly greater efficacy across the tested concentration range (up to 10 mg/mL). Quantitative analysis revealed the superior potency of HDNR-E, demonstrating a significantly lower IC_50_ value (7.94 mg/mL) compared to HDNR-W (31.22 mg/mL), indicating enhanced free radical neutralization capabilities. The ABTS assay results supported these findings, revealing significant differences in antioxidant efficacy at equivalent concentrations. Specifically, HDNR-E exhibited an IC_50_ value of 3.26 mg/mL, whereas HDNR-W had an IC_50_ value of 6.08 mg/mL, as illustrated in Figure 3B. The FRAP assay indicated that HDNR-E displayed a higher FRAP value in a concentration-dependent manner compared to HDNR-W (Figure 3C). Furthermore, HDNR-E demonstrated an IC_50_ value of 2.77 mg/mL in the FRAP assay, indicating greater reducing power compared to HDNR-W’s IC_50_ of 4.03 mg/mL. In terms of metal chelating activity (MCA), both HDNR-E and HDNR-W exhibited concentration-dependent inhibition of the Ferrozine–Fe^2^⁺ complex formation. However, HDNR-E demonstrated higher MCA activity than HDNR-W, as shown in Figure 3D. Specifically, HDNR-E displayed a lower IC_50_ value of 4.02 mg/mL compared to HDNR-W’s IC_50_ of 14.93 mg/mL. Overall, these results suggest that HDNR-E possesses greater antioxidant potential across all measured activities.

### 3.8. Inhibitory Activity of MAO, HMG-CoA Reductase, and α-Glucosidase in HDNR-W and HDNR-E Extracts

**Figure 4:**
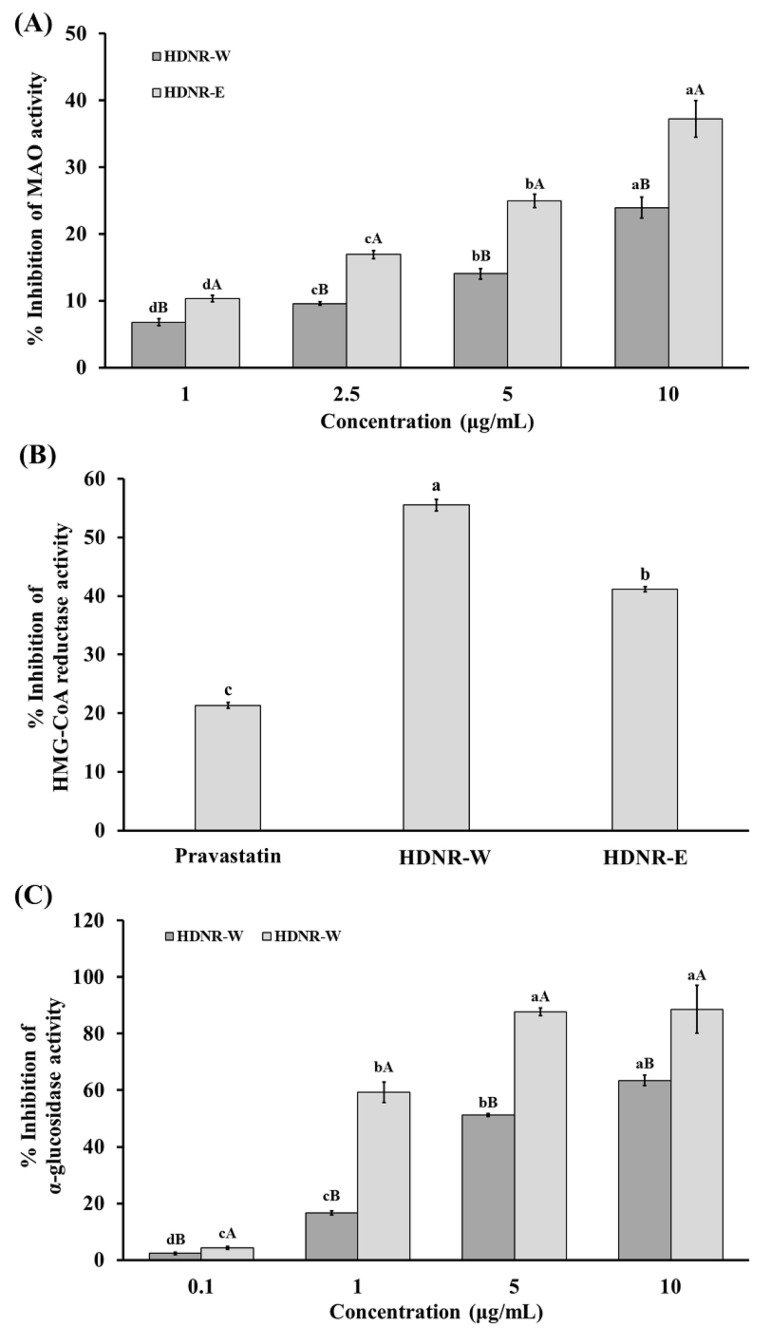
The effects of HDNR-W and HDNR-E on the inhibitory activity of (**A**) monoamine oxidase (MAO), (**B**) HMG-CoA reductase, and (**C**) α-glucosidase. Data are presented as mean ± standard deviation (*n* = 3). Pravastatin was used as a positive control for HMG-CoA reductase. Uppercase letters are used to indicate statistical significance (*p* ≤ 0.05) between HDNR-W and HDNR-E, while lowercase letters are used to denote significant differences observed at various concentrations within the same extract (*p* ≤ 0.05).

For MAO activity, HDNR-E exhibited a significantly higher inhibition rate of 37.23% at 10 mg/mL, whereas HDNR-W demonstrated an inhibition percentage of 23.94%. These results suggest that HDNR-E has a stronger effect on MAO activity (Figure 4A). In contrast, for HMG-CoA reductase activity, HDNR-W exhibited a significantly higher inhibition rate of 55.50%, while HDNR-E demonstrated an inhibition percentage of 41.14%, which was lower than that of HDNR-W. This indicates that HDNR-W has a stronger effect on HMG-CoA reductase activity (Figure 4B). Pravastatin, a positive control drug, achieved a moderate inhibition rate of 21.33% (Figure 4B). Regarding α-glucosidase activity, HDNR-E exhibited a significantly higher inhibition rate of 88.62% at 10 mg/mL, compared to HDNR-W, which demonstrated an inhibition percentage of 63.32%. These results suggest that HDNR-E has a stronger inhibitory effect on α-glucosidase activity (Figure 4C).

### 3.9. Cytotoxicity of HDNR-W and HDNR-E Extracts on Normal Cell Lines (MRC5 and hTERT-HME1) and Cancer Cell Lines (A549 and MCF7)

**Figure 5:**
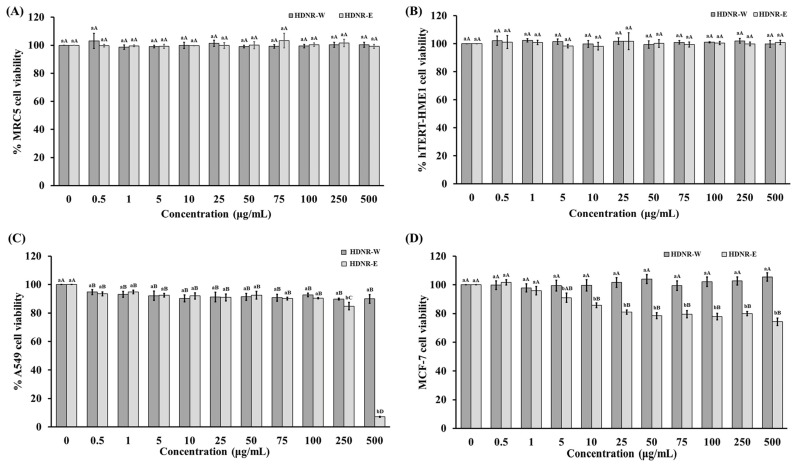
Cytotoxicity of HDNR-W and HDNR-E on (**A**) MRC5, (**B**) hTERT-HME1, (**C**) A549, and (**D**) MCF-7 cells after 24 h of treatment. The data are presented as the mean ± standard deviation (*n* = 5). Lowercase letters are used to indicate statistical significance between HDNR-W and HDNR-E at the same concentration (*p* ≤ 0.05), while uppercase letters are used to denote significant differences observed at various concentrations within the same extract (*p* ≤ 0.05).

In normal MRC5 and hTERT-HME1 cell lines, neither HDNR-W nor HDNR-E induced cytotoxicity, with cell viability remaining above 95% (Figure 5A,B). For cancer cell lines, HDNR-W did not affect the cell viability of A549 cells at any concentration. However, HDNR-E exhibited a gradual reduction in cell viability as the concentration increased, with significant reductions observed at higher concentrations (250 and 500 μg/mL), indicating potent inhibitory effects on cancer cell proliferation (Figure 5C). Additionally, HDNR-W also did not affect the cell viability of MCF-7 breast cancer cells at any concentration. Conversely, HDNR-E consistently decreased cell viability with increasing concentrations, significantly reducing viability by 25.70% at 500 μg/mL. When comparing samples at the same concentration, HDNR-W generally exhibited higher cell viability percentages than HDNR-E. At higher concentrations (250 and 500 μg/mL), HDNR-E induced significantly lower cell viability than HDNR-W, highlighting differences in potency and efficacy (Figure 5D).

## 4. Discussion

The World Health Organization has recognized NCDs as a critical development challenge of the twenty-first century. NCDs cause over 41 million deaths annually, with more than 15 million of these deaths occurring prematurely, predominantly among individuals aged 30 to 69. The primary contributors to NCD mortality include cardiovascular diseases, cancers, chronic respiratory diseases, and diabetes [1,2]. Recently, there has been an increased interest in local rice types, largely due to a growing preference for organic and healthier food options [22]. Hawm Gra Dang Ngah rice is particularly famous for its unique floral scent and subtly sweet taste, making it popular in many dishes. This rice serves as a significant source of energy and is rich in essential vitamins and minerals [4]. The aim of this study was to compare the levels of key compounds, such as phenolics, flavonoids, and anthocyanins, as well as the biological activities of HDNR-W and HDNR-E.

Water and ethanolic extraction are commonly employed techniques for isolating compounds from plant materials, each providing distinct advantages due to their differing polarities and interactions with bioactive compounds [23]. These findings highlight the efficacy of the ethanolic extraction method (HDNR-E) for isolating bioactive compounds, such as phenolics, flavonoids, and anthocyanins, from this rice material compared to water extraction (HDNR-W). The results were consistent with the LC-MS/MS analysis, which identified a greater presence of various types of phenolics, flavonoids, and anthocyanins in HDNR-E than in HDNR-W. Ethanol’s superior ability to solubilize these compounds possibly explains the higher concentrations observed in the ethanolic extracts. In contrast, water’s versatility allows it to effectively extract hydrophilic substances [23]. This observation was consistent with previous studies that examined the impact of various extraction solvents on the bioactive content of plant extracts [24,25] and highlighted that ethanol’s ability to form hydrogen bonds with phenolic compounds enhances their solubility, resulting in improved extraction yields of these compounds. Similarly, the increased total flavonoid and anthocyanin content observed in the ethanolic extracts is consistent with findings from Chaves et al. [25] and Shi et al. [26], which indicated that ethanol extraction produced significantly higher levels of flavonoids and anthocyanins compared to other solvents, including water. Taken together, these related studies support our findings, demonstrating that the ethanolic extraction method is more efficient in extracting a variety of bioactive compounds, including phenolics, flavonoids, and anthocyanins, from plant materials.

The comparison of HDNR-W and HDNR-E revealed significant differences in their amino acid profiles, highlighting the influence of solvent selection on extraction efficiency from rice samples. Both extracts contained essential and non-essential amino acids; however, HDNR-W exhibited a greater concentration of both categories. Notably, hydroxyproline, hydroxylysine, and proline were present exclusively in HDNR-W and absent in HDNR-E. Additionally, HDNR-W had a higher total content of hydrophilic amino acids (HPLs) and hydrophobic amino acids (HPBs). Water’s strong affinity for amino acids, facilitated by hydrogen bonding with functional groups such as amino and carboxyl groups, enhances extraction yields compared to ethanol, which interacts less effectively with polar residues [27,28]. Both extracts also exhibited distinct concentrations of non-protein amino acids (NPAAs), further emphasizing the varying extraction efficiencies and potential health benefits associated with compounds like taurine and GABA [29]. Studies on brown rice protein isolates have shown a high total amino acid content of approximately 78%, comprising 36% essential amino acids and 18% branched-chain amino acids [30]. The extraction method significantly influences the amino acid composition and concentration in rice extracts [27,28]. This study suggests that water extraction is more effective than ethanol, as HDNR-W consistently demonstrated higher levels of individual amino acids due to its polar nature.

A comprehensive analysis of the fatty acid composition identified and characterized the fatty acids present in HDNR-W and HDNR-E. The results revealed that both extracts do not differ in three distinct categories of fatty acids: saturated fatty acids (SFA), monounsaturated fatty acids (MUFA), and polyunsaturated fatty acids (PUFA). Water extraction primarily targets polar compounds [28], which limits its effectiveness in extracting non-polar fatty acids. In contrast, ethanol extraction offers greater versatility and higher yields, particularly for long-chain and polyunsaturated fatty acids [31]. The FT-IR analysis effectively identified the functional groups and chemical bonds present in HDNR-W and HDNR-E. Distinct spectral characteristics emerged due to the different polarities and extraction efficiencies of the solvents used. The presence of flavonoids, phenolics, and anthocyanins was evident in specific spectral regions associated with carbonyl (C=O) stretching, hydroxyl (-OH) groups, aromatic rings, and carbohydrates [32]. Bands in the carbonyl stretching region (1700–1750 cm^−1^) indicated the presence of carbonyl-containing compounds, such as fatty acids and amines [33], which were found in both samples. Variations in band intensities reflected differences in extraction efficiency. In the hydroxyl group region (3200–3600 cm^−1^), HDNR-W displayed strong bands, whereas HDNR-E exhibited varying intensities due to ethanol’s interactions with these groups. These findings suggest the presence of hydroxyl-containing compounds, including alcohols, phenols, and sugars [34]. The distinct spectral features observed in the higher wavenumber range of 3000–2800 cm^−1^, characterized by pronounced triplet bands, can be attributed to the C-H stretching vibrations originating from the methylene and methyl groups present in lipid backbones [34]. The differences in band intensities imply variations in the lipid composition extracted by each solvent. In the aromatic ring region, bands at 1600, 1512, and 1429 cm^−1^ in both samples suggest the presence of aromatic compounds, such as phenols and flavonoids [32]. These findings are in agreement with Alara et al. [24], which emphasized ethanol’s superior solubility for bioactive molecules. Overall, the variations in FT-IR spectra were attributed to the differential solubility and interactions of compounds with the solvents used.

Medicinal plants are widely recognized for their potential health benefits, particularly due to their antioxidant properties. Antioxidants play a vital role in neutralizing free radicals, thereby protecting cells from oxidative stress and contributing to overall health. [35]. The findings of this study indicate that the antioxidant activity of HDNR-E was higher than that of HDNR-W, as measured by DPPH, ABTS, FRAP, and MCA assays. Additionally, our results indicated that HDNR-E exhibited significantly lower IC_50_ values for antioxidant activity compared to the water extracts. Several studies have investigated the influence of extraction methods on antioxidant activity and reported findings consistent with the results. Mohammed et al. [36] attributed the enhanced antioxidant activity observed in ethanolic extracts to ethanol’s ability to dissolve a wider range of bioactive compounds from plant material. Similarly, studies by Kaur and Ubeyitogullari [37] and Wanyo et al. [38] highlighted ethanol’s capacity to extract phenolics and flavonoids—compounds known for their potent antioxidant properties. Previous studies have also demonstrated ethanol’s effectiveness in extracting polyphenols and other organic compounds with metal-chelating properties [39,40], which likely contributes to the higher MCA observed in ethanolic extracts. This enhanced extraction efficiency is believed to result from ethanol’s solubility characteristics, enabling it to recover a broader array of bioactive compounds. Consequently, ethanolic extracts exhibit stronger radical-scavenging capabilities and greater metal-chelating activity. The collective evidence from these studies supports the conclusion that ethanolic extraction is a preferred method for obtaining rice extracts with enhanced antioxidant activity. The higher presence of bioactive compounds, particularly phenolics and flavonoids, in ethanolic extracts can be attributed to ethanol’s superior solubility properties [24,37,38,39,40]. These findings underscore the importance of selecting appropriate solvents for maximizing the recovery of antioxidants from medicinal plants.

Monoamine oxidase (MAO) inhibitors derived from plants have garnered considerable attention for their therapeutic potential in mood regulation and neuroprotection. MAO enzymes, which metabolize neurotransmitters such as serotonin, dopamine, and phenylethylamine, play a critical role in maintaining neurological function. Various plants have been shown to inhibit MAO activity, thereby influencing neurotransmitter levels and offering promising applications for the treatment of neuropsychiatric and neurodegenerative diseases [41]. Recent findings indicate that HDNR-E exhibits higher inhibitory activity against MAO compared to HDNR-W, suggesting its enhanced efficacy in modulating neurotransmitter levels. This activity may be attributed to the presence of bioactive compounds such as flavonoids and phenolic acids, which are known to act as natural MAO inhibitors due to their structural similarity to synthetic inhibitors. For example, plant extracts like green tea and citrus peels have shown potent MAO-inhibitory effects [42,43]. Similarly, alpha-glucosidase inhibitors are widely used in diabetes management due to their ability to delay carbohydrate digestion and absorption in the intestine, thereby reducing postprandial blood glucose spikes. Synthetic inhibitors such as acarbose and miglitol are effective but often associated with gastrointestinal side effects [18,44]. Natural alternatives are increasingly recognized for their comparable efficacy and reduced adverse effects [45]. Our findings revealed that HDNR-E exhibited higher inhibitory activity against alpha-glucosidase compared to HDNR-W, suggesting the presence of potent bioactive compounds. Supporting evidence shows that methanolic extracts of black rice bran achieve up to 62% alpha-glucosidase inhibition, while phenolic compounds such as methyl vanillate, syringic acid, and vanillic acid from Thai-colored rice cultivars exhibit strong inhibitory effects [46,47]. The phenolic compounds present in these natural extracts not only mimic the structural properties of synthetic inhibitors but also provide additional health benefits due to their antioxidant properties. The higher concentration of bioactive compounds, particularly phenolics and flavonoids, in ethanolic extracts like HDNR-E supports its dual inhibitory activity against both MAO and alpha-glucosidase. Furthermore, several herbal extracts have demonstrated potential in inhibiting HMG-CoA reductase, a crucial enzyme targeted by cholesterol-lowering therapies [11,48,49]. Notably, HDNR-W exhibited substantial inhibition of HMG-CoA reductase, indicating a more potent effect on reducing cholesterol synthesis compared to HDNR-E. Our previous studies have shown that the water extract possesses a higher potency in inhibiting HMG-CoA reductase than the ethanolic extract [49]. This difference in inhibitory activity may be attributed to the distinct phytochemical compositions of these extracts, including their total phenolic, flavonoid, and anthocyanin content, as well as their amino acid and fatty acid profiles [11,48]. These bioactive compounds are known to modulate enzyme activity and contribute to the overall therapeutic effects of herbal extract [48,49]. Additionally, certain amino acids, such as lysine and arginine, have been reported to inhibit HMG-CoA reductase activity [50]. This suggests that the amino acid profile of HDNR-W could play a significant role in their cholesterol-lowering effects. The structural diversity of phytochemicals in these extracts allows them to interact with the active site of HMG-CoA reductase, thereby reducing cholesterol synthesis in the liver.

Several studies have identified various plants and their bioactive compounds that demonstrate cytotoxic effects against different cancer cell lines [45]. Our findings indicate that HDNR-E, at high concentrations, reduced cancer cell proliferation in both A549 and MCF-7 cells. In contrast, neither HDNR-W nor HDNR-E affected normal cells. These effects might be attributed to the high levels of total phenolics, flavonoids, and anthocyanins present in the samples, which may exert antioxidant effects and modulate signaling pathways involved in cell proliferation and apoptosis [51]. Additionally, the amino acid composition, particularly the presence of arginine and glutamine, may influence cancer cell metabolism and proliferation [52]. Specific amino acids could inhibit cancer cell growth by interfering with essential metabolic pathways [53]. Furthermore, the fatty acid profile, including polyunsaturated fatty acids like omega-3s, is associated with anti-cancer activity due to their role in modulating inflammation and inhibiting cancer cell proliferation [54]. Overall, these bioactive compounds likely contribute to the observed inhibition of cancer cell growth in A549 and MCF-7 cells while remaining non-toxic to normal cells.

## 5. Conclusions

HDNR-E outperformed HDNR-W in extracting total phenolics, flavonoids, and anthocyanins, which can be attributed to ethanol’s superior solubilization capacity. The FT-IR analysis revealed distinct peak intensities and patterns, indicating diverse interactions between the rice extract and solvents. Specifically, variations were observed in the carbonyl stretching, hydroxyl group, C-H stretching vibration, and aromatic ring regions, reflecting solvent-dependent solubility and compound interactions. In the carbohydrate region, HDNR-W and HDNR-E exhibited potential compositional differences. These variations in band intensities were attributed to differential solubility, polarity, and solvent interactions. The antioxidant activity demonstrated that HDNR-E consistently exhibited higher DPPH, ABTS, and MCA, as well as superior FRAP values and lower IC_50_ values, highlighting its enhanced antioxidant potential. Additionally, both extracts significantly inhibited MAO, α-glucosidase, and HMG-CoA reductase activities. Importantly, HDNR-E inhibited the proliferation of A549 and MCF-7 cancer cells without affecting normal cells, suggesting its greater potential as an anticancer agent compared to HDNR-W. Collectively, the results highlight the importance of solvent selection in extracting diverse bioactive compounds, which influences both spectral attributes and biological activities. These results indicate that HDNR-E holds promise for further biological studies. However, additional studies on bioavailability, as well as in vitro and in vivo investigations, are needed to evaluate its effectiveness.

## Figures and Tables

**Table 1:** The chemical constituents in HDNR-W by UPLC-MS/MS.

No.	RT (min)	Identification	Molecular Formula	Experimental Mass (*m*/*z*)	Error (ppm)	Category
**Negative mode**						
1	1.621	Phytic acid	C_6_H_18_O_246_	658.854	0.38	Phosphorus Compound
2	1.646	myo-Inositol pentakisphosphate	C_6_H_17_O_21_P_5_	578.8874	0.76	Phosphorus Compound
3	1.696	1D-myo-Inositol 1,3,4,6-tetrakisphosphate	C_6_H_16_O_18_P_4_	498.9212	0.58	Phosphorus Compound
4	1.822	L-Iditol	C_6_H_14_O_6_	181.0713	2.7	Sugar Alcohol
5	1.897	D-Mannonate	C_6_H_12_O_7_	195.051	0.43	Sugar Acid
6	1.922	L-Xylonate	C_5_H_10_O_6_	165.0401	2.11	Sugar Acid
7	2.047	Sucrose	C_12_H_22_O_11_	341.1082	1.43	Disaccharide
8	2.085	Glyceric acid	C_3_H_6_O_4_	105.0189	3.87	Sugar Acid
9	2.26	Malic acid	C_4_H_6_O_5_	133.0138	3.22	Organic Acid
10	2.423	Sarmentosin epoxide	C_11_H_17_NO_8_	290.0872	3.17	Alkaloid
11	2.523	Hypoxanthine	C_5_H_4_N_4_O	135.0307	3.44	Purine Derivative
12	2.649	Citric acid	C_6_H_8_O_7_	191.0199	−0.45	Organic Acid
13	2.673	2,3-Dihydroxy-2,4-cyclopentadien-1-one	C_5_H_4_O_3_	111.0085	2.44	Phenolic Compound
14	2.786	Pyroglutamic acid	C_5_H_7_NO_3_	128.035	2.48	Amino Acid Derivative
15	2.849	Pseudouridine	C_9_H_12_N_2_O_6_	243.0617	2.14	Nucleoside
16	3.024	Maleic acid	C_4_H_4_O_4_	115.0034	2.29	Organic Acid
17	3.049	Meta-Tyrosine	C_9_H_11_NO_3_	180.0661	1.87	Amino Acid Derivative
18	3.275	(R)-2,3-Dihydro-3,5-dihydroxy-2-oxo-3-indoleacetic acid	C_10_H_9_NO_5_	222.04	3.36	Alkaloid
19	4.102	DIMBOA-Glc	C_15_H_19_NO_10_	372.0926	2.7	Alkaloid Glycoside
20	4.666	L-Phenylalanine	C_9_H_11_NO_2_	164.0711	3.16	Amino Acid
21	4.929	4-Hydroxyisoleucine	C_6_H_13_NO_3_	146.0819	2.33	Amino Acid
22	4.954	Pantothenic Acid	C_9_H_17_NO_5_	218.1036	−0.86	Vitamin
23	5.706	6-Caffeoylsucrose	C_21_H_28_O_14_	503.1399	1.72	Phenylpropanoid Glycoside
24	5.831	Mandelonitrile sophoroside	C_20_H_27_NO_11_	456.1502	2.03	Cyanogenic Glycoside
25	5.931	3,4-Dihydroxybenzoic acid	C_7_H_6_O_4_	153.0191	1.74	Phenolic Acid
26	5.956	Pyrocatechol	C_6_H_6_O_2_	109.0293	2.05	Phenolic Compound
27	6.307	L-Tryptophan	C_11_H_12_N_2_O_2_	203.082	3.04	Amino Acid
28	6.933	Procyanidin B2	C_30_H_26_O_12_	577.1345	1.4	Flavonoid
29	6.933	Epigallocatechin 3-O-(4-hydroxybenzoate)	C_22_H_18_O_9_	425.087	2.25	Flavonoid
30	7.209	Potassium 2-(1′-ethoxy) ethoxypropanoate	C_7_H_144_	161.0815	2.85	Organic Salt
31	7.334	Epicatechin	C_15_H_14_O_6_	289.071	2.55	Flavonoid
32	7.359	3,4-Dihydroxybenzaldehyde	C_7_H_6_O_3_	137.0241	2.23	Phenolic Compound
33	8.261	Phloracetophenone	C_8_H_8_O_4_	167.0346	2.48	Phenolic Compound
34	8.311	Bisbynin	C_15_H_22_O_5_	281.1386	3.27	Sesquiterpenoid
35	8.524	APIIN	C_26_H_28_O_14_	563.1395	2.03	Flavonoid Glycoside
36	8.913	Dihydroferulic acid 4-O-glucuronide	C_16_H_20_O_10_	371.0977	2.13	Phenylpropanoid Glycoside
37	9.965	Phloroacetophenone 6′-[xylosyl-(1->6)-glucoside]	C_21_H_30_O_13_	489.1606	1.86	Phenolic Glycoside
38	9.99	Lentialexin	C_8_H_8_O	119.05	2.53	Phenolic Compound
39	10.441	Sinapic acid	C_11_H_12_O_5_	223.0605	2.99	Phenylpropanoid
40	10.579	Scytalone	C_10_H_10_O_4_	193.0499	3.79	Phenolic Compound
41	12.32	p-Salicylic acid	C_7_H_6_O_3_	137.0242	1.92	Phenolic Acid
42	12.872	9S,10S,11R-trihydroxy-12Z-octadecenoic acid	C_18_H_34_O_5_	329.2331	0.03	Fatty Acid
43	13.347	Isoleptospermone	C_15_H_22_O_4_	265.1435	3.73	Sesquiterpenoid
44	13.724	Myrsinone	C_17_H_26_O_4_	293.1753	1.98	Diterpenoid
45	13.773	Thyrotropin-releasing hormone	C_16_H_22_N_6_O_4_	361.1626	0.92	Peptide Hormone
46	14.074	(±)12,13-DiHOME	C_18_H_34_O_4_	313.2381	1.05	Fatty Acid
47	14.112	Val His Lys	C_17_H_30_N_6_O_4_	381.2249	1.47	Tripeptide
48	14.726	13(S)-HODE	C_18_H_32_O_3_	295.2272	2.51	Fatty Acid
49	19.098	Vaccenic acid	C_18_H_34_O_2_	281.2477	2.76	Fatty Acid
**Positive mode**						
1	1.897	Betaine	C_5_H_12_NO_2_	118.0866	−2.79	Quaternary Ammonium Compound
2	1.997	Sucrose	C_12_H_22_O_11_	365.1057	−1.76	Carbohydrate
3	2.072	Cytosine	C_4_H_5_N_3_O	112.0508	−2.79	Nucleobase
4	2.147	Epidermin	C_11_H_19_NO_6_	262.1285	0.31	Lipopeptide
5	2.348	Niacin (Nicotinic acid)	C_6_H_5_NO_2_	124.0399	−4.53	Vitamin (B-complex)
6	2.473	Pro Leu	C_11_H_20_N_2_O_3_	229.1554	−2.92	Dipeptide
7	2.523	Hypoxanthine	C_5_H_4_N_4_O	137.0462	−3.61	Purine Derivative
8	2.548	N-Methylanthranilic Acid	C_8_H_9_NO_2_	152.0709	−2.64	Alkaloid
9	2.724	(S)-2,3-Dihydro-3,5-dihydroxy-2-oxo-3-indoleacetic acid 5-glucoside	C_16_H_19_NO_10_	408.0902	−0.02	Indole Glucoside
10	2.824	Pyroglutamic acid	C_5_H_7_NO_3_	130.0498	0.71	Amino Acid Derivative
11	2.924	Adenosine	C_10_H_13_N_5_O_4_	268.1045	−0.36	Nucleoside
12	3.037	Pirbuterol	C_12_H_20_N_2_O_3_	241.1549	−1.12	Beta-Agonist
13	3.1	Guanine	C_5_H_5_N_5_O	152.0572	−3.68	Nucleobase
14	3.325	Pirbuterol	C_12_H_20_N_2_O_3_	241.1548	−0.6	Benzoxazinoid
15	3.551	Pirbuterol	C_12_H_20_N_2_O_3_	241.1548	−0.58	Vitamin (B-complex)
16	4.002	DIMBOA-Glc	C_15_H_19_NO_10_	374.1082	0.04	Aminobenzoic Acid Derivative
17	4.803	Pantothenic Acid	C_9_H_17_NO_5_	242.1004	−1.89	Flavonoid
18	6.282	3-Amino-2-naphthoic acid	C_11_H_9_NO_2_	188.0711	−2.28	Polysaccharide
19	6.808	Procyanidin B2	C_30_H_26_O_12_	579.1491	1.19	Alkaloid
20	7.083	Galactan	C_20_ H_36_O_16_	571.1626	1.07	Phenylpropanoid
21	9.564	Gelsedine	C_19_H_24_ N_2_ O_3_	346.2128	−0.8	Alkaloid
22	10.516	3-(3,4-Methylenedioxyphenyl)propenal	C_10_H_8_O_3_	177.0547	−1.07	Tripeptide
23	10.541	Compound IV	C_20_H_26_N_2_O_3_	360.2287	−1.78	Aminoglycoside
24	13.084	His Gln Glu	C_16_H_24_N_6_O_7_	413.1784	−1.13	Tripeptide
25	13.159	1-O-[2-(Acetylamino)-2-deoxy-alpha-D-glucopyranosyl]-D-myo-Inositol	C_14_H_25_NO_11_	401.1782	−4.58	Peptide Toxin
26	13.222	Asn His Gly	C_12_H_18_N_6_O_5_	327.1419	−3.03	Sphingolipid
27	13.272	HC Toxin	C_21_H_32_N_4_O_6_	459.2199	3.23	Sphingolipid
28	13.323	Phytosphingosine	C_18_H_39_NO_3_	318.301	−1.76	Tripeptide
29	13.372	C16 Sphinganine	C_16_H_35_NO_2_	274.2741	−0.11	Tripeptide
30	13.423	Gln Phe Met	C_19_H_28_N_4_O_5_S	425.1849	0.93	Sphingolipid
31	13.448	Thr His Gln	C_15_H_24_N_6_O_6_	385.1841	−3.31	Hydroxy Fatty Acid
32	13.523	C17 Sphinganine	C_17_H_37_NO_2_	288.2898	−0.43	Cytokinin Derivative
33	13.748	(3S,4S)-3-hydroxytetradecane-1,3,4-tricarboxylic acid	C_17_H_30_O_7_	369.1884	0.19	Phosphonoglycine
34	13.924	trans-Zeatin-O-glucoside riboside	C_21_H_31_N_5_O_10_	531.2409	−0.34	Tripeptide
35	14.187	N-Acetylbialaphos	C_13_H_24_N_3_O_7_P	383.1684	1.33	Fatty Acid Amide
36	14.387	Tyr Asn Gln	C_18_H_25_N_5_O_7_	441.2094	−1.22	Ester
37	14.75	dodecanamide	C_12_H_25_NO	200.2011	−1.07	Fatty Acid Amide
38	15.327	Dibutyl phthalate	C_16_H_22_O_4_	301.1421	−3.45	Fatty Acid Amide
39	16.442	13E-Docosenamide	C_22_H_43_NO	338.3422	−1.14	Bile Acid
40	19.085	N-stearoyl valine	C_23_H_45_NO_3_	406.3288	0.99	Aminophenone Derivative
41	22.355	6β-Hydroxy-3-oxo-5β-cholan-24-oic Acid	C_24_H_38_O_4_	413.266	0.67	Quaternary Ammonium Compound
42	22.568	2-(ethylamino)-4′-hydroxy-Propiophenone	C_11_H_15_NO_2_	194.1175	0.3	Carbohydrate

**Table 2:** The chemical constituents in HDNR-E by UPLC-MS/MS.

No.	RT (min)	Identification	Molecular Formula	Experimental Mass (*m*/*z*)	Error (ppm)	Category
**Negative mode**						
1	1.865	D-Sorbitol	C_6_H_14_O_6_	181.0714	2.19	Sugar Alcohol
2	2.028	Sucrose	C_12_H_22_O_11_	341.1091	−0.43	Carbohydrate
3	2.04	Glyceric acid	C_3_H_6_O_4_	105.019	3.04	Organic Acid
4	2.241	Pyroglutamic acid	C_5_H_7_NO_3_	128.0351	1.62	Amino Acid Derivative
5	2.892	2,3-Dihydroxy-2-methylbutanoic acid	C_5_H_10_O_4_	133.0504	3.97	Organic Acid
6	4.809	Pantothenic Acid	C_9_H_17_NO_5_	218.1029	2.17	Vitamin (B-complex)
7	5.874	3,4-Dihydroxybenzoic acid	C_7_H_6_O_4_	153.0192	0.93	Phenolic Acid
8	5.899	Pyrocatechol	C_6_H_6_O_2_	109.0293	1.51	Phenol
9	6.701	Procyanidin B2	C_30_H_26_O_12_	577.1349	0.74	Flavonoid
10	6.851	Epigallocatechin 3-O-(4-hydroxybenzoate)	C_22_H_18_O_9_	425.0867	2.48	Flavonoid
11	6.914	Cinnamtannin A1	C_45_H_38_O_18_	865.197	2.04	Flavonoid (Tannin)
12	7.264	Epicatechin	C_15_H_14_O_6_	289.0716	0.86	Flavonoid
13	7.352	3,4-Dihydroxybenzaldehyde	C_7_H_6_O_3_	137.0244	0.38	Phenolic Aldehyde
14	8.329	Bisbynin	C_15_H_22_O_5_	281.1387	2.81	Phenolic Compound
15	8.705	APIIN	C_26_H_28_O_14_	563.1397	1.9	Flavonoid Glycoside
16	9.958	Phloroacetophenone 6′-[xylosyl-(1->6)-glucoside]	C_21_H_30_O_13_	489.1602	2.26	Phenolic Glycoside
17	9.958	Lentialexin	C_8_H_8_O	119.0501	1.48	Phenylpropanoid
18	10.434	Sinapic acid	C_11_H_12_O_5_	223.0607	2.33	Phenolic Acid
19	10.572	Scytalone	C_10_H_10_O_4_	193.0498	4.31	Phenolic Compound
20	10.584	Phloridzin	C_21_H_24_O_10_	435.1281	3.66	Flavonoid Glycoside
21	11.01	Natsudaidain 3-(4-O-3-hydroxy-3-methylglutaroylglucoside)	C_33_H_40_O_18_	723.2127	2.3	Phenolic Glycoside
22	12.313	p-Salicylic acid	C_7_H_6_O_3_	137.0242	1.84	Phenolic Acid
23	12.514	Luteolin	C_15_H_10_O_6_	285.0397	2.54	Flavonoid
24	12.802	9,10,13-Trihydroxystearic acid	C_18_H_36_O_5_	331.2484	2.09	Hydroxy Fatty Acid
25	12.865	9S,10S,11R-trihydroxy-12Z-octadecenoic acid	C_18_H_34_O_5_	329.2336	−0.31	Hydroxy Fatty Acid
26	12.964	Diosmetin	C_16_H_12_O_6_	299.0555	2.07	Flavonoid
27	13.015	9,10-dihydroxy-hexadecanoic acid	C_16_H_32_O_4_	287.2224	1.38	Hydroxy Fatty Acid
28	13.34	N-Oleoyl-L-Serine	C_21_H_39_NO_4_	368.28	1.63	Fatty Acid Amide
29	13.441	Obliquine	C_26_H_28_N_2_O_5_	447.1921	0.92	Alkaloid
30	13.566	Lipomycin	C_32_H_45_NO_9_	586.2997	4.59	Polyketide
31	13.716	Myrsinone	C_17_H_26_O_4_	293.175	2.74	Phenolic Compound
32	13.766	S-cucujolide V	C_14_H_22_O_2_	221.1538	4	Macrolide Compound
33	13.967	LysoPE(0:0/14:0)	C_19_H_40_NO_7_P	424.2465	1.26	Lysophospholipid
34	14.142	(±)12,13-DiHOME	C_18_H_34_O_4_	313.2382	0.84	Hydroxy Fatty Acid
35	14.418	9,14-dihydroxy-octadecanoic acid	C_18_H_36_O_4_	315.253	3.56	Hydroxy Fatty Acid
36	14.455	PE(18:2(9Z,12Z)/0:0)	C_23_H_44_NO_7_P	476.2774	2.35	Phospholipid
37	14.543	8-HpODE	C_18_H_32_O_4_	311.2229	−0.09	Hydroxy Fatty Acid
38	14.769	13(S)-HODE	C_18_H_32_O_3_	295.2275	1	Hydroxy Fatty Acid
39	14.919	(S)-Nerolidol 3-O-[a-L-rhamnopyranosyl-(1->2)-b-D-glucopyranoside]	C_27_H_46_O_10_	529.3019	0.38	Glycoside
40	15.019	Ricinoleic acid	C_18_H_34_O_3_	297.2431	1.78	Hydroxy Fatty Acid
41	16.046	cholesterol sulfate	C_27_H_46_O_4_S	465.3063	−4.06	Steroid Sulfate
42	16.948	cis-9,10-Epoxystearic acid	C_18_H_34_O_3_	297.2427	2.94	Epoxy Fatty Acid
43	17.324	10E,12Z-Octadecadienoic acid	C_18_H_32_O_2_	279.2321	2.91	Unsaturated Fatty Acid
44	18.276	19-hydroxy-nonadecanoic acid	C_19_H_38_O_3_	313.2741	2.61	Hydroxy Fatty Acid
45	18.715	DL-2-hydroxy stearic acid	C_18_H_36_O_3_	299.2587	1.8	Hydroxy Fatty Acid
46	18.74	PG(18:2(9Z,12Z)/0:0)	C_24_H_45_O_9_P	507.2726	0.91	Phospholipid
47	19.053	Vaccenic acid	C_18_H_34_O_2_	281.2482	1.33	Unsaturated Fatty Acid
48	19.291	Elaidic Acid	C_18_H_34_O_2_	281.2484	0.86	Unsaturated Fatty Acid
49	21.458	15-methoxy-tricosanoic acid	C_24_H_48_O_3_	383.3523	2.19	Methoxy Fatty Acid
50	22.336	PG(16:0/0:0)	C_22_H_45_O_9_P	483.2726	0.65	Phospholipid
**Positive mode**						
1	1.887	Betaine	C_5_H_12_NO_2_	118.0866	−3.36	Quaternary Ammonium Compound
2	1.974	Sucrose	C_12_H_22_O_11_	365.1057	−1.05	Disaccharide
3	3.39	Pro Leu	C_11_H_20_N_2_O_3_	229.1552	−1.75	Dipeptide
4	6.197	3-Amino-2-naphthoic acid	C_11_H_9_NO_2_	188.0709	−1.36	Amino Acid Derivative
5	6.698	Procyanidin B2	C_30_H_26_O_12_	579.1492	1.12	Flavonoid
6	7.073	Galactan	C_20_H_36_O_16_	571.163	0.64	Polysaccharide
7	9.103	Ceanothine E	C_34_H_40_N_4_O_4_	569.3138	−2.73	Alkaloid
8	9.554	Gelsedine	C_19_H_24_N_2_O_3_	346.2129	−1.08	Alkaloid
9	9.905	Coumarin	C_9_H_6_O_2_	147.044	−0.23	Benzopyrone
10	10.381	Compound IV	C_20_H_26_N_2_O_3_	360.2288	−2.47	Alkaloid
11	10.456	Eugenin	C_11_H_10_O_4_	207.0653	−0.67	Phenolic Compound
12	12.072	Pro Asp Arg	C_15_H_26_N_6_O_6_	387.1991	−1.34	Tripeptide
13	12.21	Quinoline	C_9_H_7_N	130.0653	−3.54	Heterocyclic Compound
14	12.36	Patuletin 3-rhamnoside-7-(3′′′,4′′′-diacetylrhamnoside)	C_32_H_36_O_18_	709.194	4.96	Flavonoid Glycoside
15	12.41	Berberine	C_20_H_18_NO_4_	336.1231	−0.03	Alkaloid
16	12.811	6-Epi-7-isocucurbic acid glucoside	C_18_H_30_O_8_	397.1836	−0.64	Glycoside
17	12.836	HC Toxin	C_21_H_32_N_4_O_6_	459.22	2.18	Cyclic Tetrapeptide
18	12.861	9S,10S,11R-trihydroxy-12Z-octadecenoic acid	C_18_H_34_O_5_	353.2301	−0.42	Hydroxy Fatty Acid
19	13.012	Leu Lys Asp	C_16_H_30_N_4_O_6_	413.1786	−0.06	Tripeptide
20	13.061	Glucosyl (E)-2,6-Dimethyl-2,5-heptadienoate	C_15_H_24_O_7_	339.1415	−0.18	Glycoside
21	13.212	C16 Sphinganine	C_16_H_35_NO_2_	274.2748	−2.57	Sphingolipid
22	13.362	Thr His Gln	C_15_H_24_N_6_O_6_	385.1835	−1.79	Tripeptide
23	13.538	Dehydrophytosphingosine	C_18_H_37_NO_3_	316.2851	−1.54	Sphingolipid
24	13.613	PI(16:1(9Z)/0:0)	C_25_H_47_O_12_P	588.3145	−0.02	Phosphatidylinositol
25	13.763	Glucosyl sphingosine	C_24_H_47_NO_7_	462.3431	−1.36	Glycosphingolipid
26	13.776	Linoleamide	C_18_H_33_NO	280.2641	−1.83	Fatty Acid Amide
27	13.801	(Z)-N-(2-hydroxyethyl)hexadec-7-enamide	C_18_H_35_NO_2_	298.275	−2.92	Amide Compound
28	13.814	Phytosphingosine	C_18_H_39_NO_3_	318.301	−2.14	Sphingolipid
29	13.914	Asp Phe Trp	C_24_H_26_N_4_O_6_	467.1946	−4.71	Tripeptide
30	13.939	6-Hydroxyfluvastatin	C_24_H_26_FNO_5_	445.2129	1.24	Statin
31	14.001	PI(20:3(8Z,11Z,14Z)/0:0)	C_29_H_51_O_12_P	640.346	−0.44	Phosphatidylinositol
32	14.114	Buprenorphine	C_29_H_41_NO_4_	468.3103	1.74	Opioid
33	14.151	PC(18:3(6Z,9Z,12Z)/0:0)	C_26_H_49_NO_7_P	518.3247	−1.01	Phosphatidylcholine
34	14.239	PE(17:0/0:0)	C_22_H_46_NO_7_P	490.2911	−1.14	Phosphatidylethanolamine
35	14.364	Sphinganine	C1_8_H_39_NO_2_	302.306	−0.74	Sphingolipid
36	14.49	Dihydroceramide C2	C_20_H_41_NO_3_	344.3169	−2.76	Sphingolipid
37	14.565	PI(18:1(9Z)/0:0)	C_27_H_51_O_12_P	616.3462	−0.73	Phosphatidylinositol
38	14.59	PE(18:2(9Z,12Z)/0:0)	C_23_H_44_NO_7_P	478.2935	−1.28	Phosphatidylethanolamine
39	15.943	Linoleoyl Ethanolamide	C_20_H_37_NO_2_	324.2906	−2.58	Fatty Acid Amide
40	16.131	SLF	C_30_H_40_N_2_O_6_	542.3224	−0.06	Sphingolipid
41	16.218	20:2(5Z,9Z)(11Me,15Me,19Me)	C_23_H_42_O_2_	368.3528	−1.28	Fatty Acid Derivative
42	17.17	Oleoyl Ethanolamide	C_20_H_39_NO_2_	326.3056	−0.88	Fatty Acid Amide
43	17.434	PE(19:0/0:0)	C_24_H_50_NO_7_P	518.3225	−2.44	Phosphatidylethanolamine
44	22.382	Prostaglandin F2α-biotin	C_35_H_60_N_4_O_6_S	687.4138	−1.94	Biotinylated Compound

**Table 3:** Amino acid composition in HDNR-W and HDNR-E.

Amino Acid	Abbreviation	HPL/HPB	Amino Acid Content (mg/100 g Sample)	
			HDNR-W	HDNR-E
**Essential amino acids (EAAs)**				
Histidine	His	HPL	0.48 ^a^	0.12 ^b^
Isoleucine	Ile	HPB	0.41 ^a^	0.37 ^b^
Leucine	Leu	HPB	0.69 ^b^	0.76 ^a^
Lysine	Lys	HPL	0.93 ^a^	0.27 ^b^
Methionine	Met	HPB	0.29 ^a^	0.13 ^b^
Phenylalanine	Phe	HPB	0.43 ^b^	0.50 ^a^
Threonine	Thr	HPL	1.13 ^a^	0.62 ^b^
Tryptophan	Trp	HPB	ND	ND
Valine	Val	HPB	1.22 ^a^	0.98 ^b^
Total EAAs			5.58 ^a^	3.75 ^b^
**Non-essential amino acids (NEAAs)**				
Alanine	Ala	HPB	2.40 ^a^	1.64 ^b^
Arginine	Arg	HPL	1.01 ^a^	0.48 ^b^
Asparagine	Asn	HPL	ND	ND
Aspartic acid	Asp	HPL	3.82 ^a^	1.51 ^b^
Cystine	Cys	HPB	0.44 ^a^	0.13 ^b^
Glutamic acid	Glu	HPL	4.46 ^a^	1.99 ^b^
Glutamine	Gln	HPL	ND	ND
Glycine	Gly	HPB	2.08 ^a^	1.04 ^b^
Hydroxy proline	HyPro	HPL	0.23	ND
Hydroxylysine	HyLys	HPL	0.60	ND
Proline	Pro	HPB	0.55	ND
Serine	Ser	HPL	1.09 ^a^	0.69 ^b^
Tyrosine	Tyr	HPB	0.54 ^b^	0.56 ^a^
Total NEAAs			17.22 ^a^	8.04 ^b^
Total hydrophobic amino acids (HPBs)			9.05 ^a^	6.11 ^b^
Total hydrophilic amino acids (HPLs)			13.75 ^a^	5.68 ^b^
Nonprotein Amino Acids (NPAAs)				
Ornithine	Orn		0.05 ^a^	0.04 ^a^
Taurine	Tau		0.06 ^b^	0.10 ^a^
α-Amino-n-butyric acid	AABA		0.06	ND
β-Alanine	β-Ala		0.27 ^a^	0.18 ^b^
β-Amino isobutyric acid	BAIBA		0.05 ^a^	0.05 ^a^
γ-Amino-n-butyric acid	GABA		1.57 ^a^	0.94 ^b^
Total NPAAs			2.06 ^a^	1.31 ^b^

Data are presented as mean (*n* = 3). The standard deviation (SD) values were not displayed because they were less than one hundredth (SD ≤ 0.01). ND means “Not detected”. The lowercase letters indicate statistical significance in the same row (*p* ≤ 0.05).

**Table 4:** Fatty acid composition in HDNR-W and HDNR-E.

Fatty Acid	Fatty Acid Content (%)	
	HDNR-W	HDNR-E
Saturated fatty acid (SFA)		
C14:0	1.03	1.02
C16:0	21.87	24.23
C18:0	2.78	2.13
C20:0	ND	0.40
C21:0	ND	0.01
C22:0	ND	0.10
C24:0	ND	0.17
Monounsaturated fatty acid (MUFA)		
C18:1n9t	ND	ND
C18:1n9c	40.19	30.57
Polyunsaturated fatty acid (PUFA)		
C18:2n6c	34.13	39.83
C18:3n6	ND	ND
C18:3n3	ND	1.52
C20:2	ND	0.02
Total SFA	25.68	28.05
Total USFA *	74.32	71.95
Total MUFA	40.19	30.57
Total PUFA	34.13	41.37

Data are presented as mean (*n* = 3). The standard deviation (SD) values were not displayed because they were less than one hundredth (SD < 0.01). ND means “Not detected”. * USFA means unsaturated fatty acid.

**Table 5:** Concentrations of heavy metals in HDNR-W by ICP-OES.

Concentration (mg/kg)	As	Cd	Cr	Cu	Mn	Ni	Pb	Zn	Fe
HDNR-W	0.026	0.002	0.030	0.114	1.913	0.118	0.006	0.257	0.001

## Data Availability

The original contributions presented in this study are included in the article. Further inquiries can be directed to the corresponding authors.

## References

[1] Budreviciute A., Damiati S., Sabir D.K., Onder K., Schuller-Goetzburg P., Plakys G., Katileviciute A., Khoja S., Kodzius R. (2020). Management and prevention strategies for non-communicable diseases (NCDs) and their risk factors. Front. Public Health.

[2] (2021). Noncommunicable Diseases Country Profiles.

[3] Nguanchoo V., Balslev H., Sadgrove N.J., Phumthum M. (2023). Medicinal plants used by rural Thai people to treat non-communicable diseases and related symptoms. Heliyon.

[4] Fukagawa N.K., Ziska L.H. (2019). Rice: Importance for global nutrition. J. Nutr. Sci. Vitaminol..

[5] Ma Y., Li J., Xue Y., Xu Y., Liu C., Su D. (2023). Comprehensive improvement of nutrients and volatile compounds of black/purple rice by extrusion-puffing technology. Front. Nutr..

[6] Tapia-Hernández J.A., Del-Toro-Sánchez C.L., Cinco-Moroyoqui F.J., Juárez-Onofre J.E., Ruiz-Cruz S., Carvajal-Millan E., López-Ahumada G.A., Castro-Enriquez D.D., Barreras-Urbina C.G., Rodríguez-Felix F. (2019). Prolamins from cereal by-products: Classification, extraction, characterization and its applications in micro- and nanofabrication. Trends Food Sci. Technol..

[7] Chen X., Yang Y., Yang X., Zhu G., Lu X., Jia F., Diao B., Yu S., Ali A., Zhang H. (2022). Investigation of flavonoid components and their associated antioxidant capacity in different pigmented rice varieties. Food Res. Int..

[8] Aenglong C., Woonnoi W., Tanasawet S., Klaypradit W., Sukketsiri W. (2024). Impact of time and enzyme concentration on Sangyod rice bran hydrolysate: Phytochemicals, antioxidants, amino acids, and cytotoxicity. Rice.

[9] Hanchang W., Woonnoi W., Saetan J., Suttithumsatid W., Tanasawet S., Sanprick A., Moolsup F., Sukketsiri W. (2024). Sangyod rice extract mitigates insulin resistance in HepG2 cells and hepatic steatosis in diabetic rats via AMPK/mTOR/MAPK signaling pathways. Food Biosci..

[10] Panomwan P., Temdee W. (2021). Physical and nutritional properties of local Hawm Gra Dang Ngah Rice varieties. Curr. Res. Nutr. Food Sci..

[11] Woonnoi W., Suttithumsatid W., Muneerungsee N., Saetan J., Tanasawet S., Sukketsiri W. (2023). Sangyod rice extract inhibits adipocyte growth and differentiation via mTOR, Akt, and AMPK pathways. J. Funct. Foods.

[12] Wisetkomolmat J., Arjin C., Hongsibsong S., Ruksiriwanich W., Niwat C., Tiyayon P., Jamjod S., Yamuangmorn S., Prom-U-Thai C., Sringarm K. (2023). Antioxidant activities and characterization of polyphenols from selected oorthern Thai rice husks: Relation with seed attributes. Rice Sci..

[13] Hansakul P., Srisawat U., Itharat A., Lerdvuthisopon N. (2011). Phenolic and flavonoid contents of Thai rice extracts and their correlation with antioxidant activities using chemical and cell assays. J. Med. Assoc. Thai..

[14] Lee J., Durst R.W., Wrolstad R.E. (2019). Determination of total monomeric anthocyanin pigment content of fruit juices, beverages, natural colorants, and wines by the pH differential method: Collaborative Study. J. AOAC Int..

[15] Curtis J.M., Berrigan N., Dauphinee P. (2008). The determination of n-3 fatty acid levels in food products containing microencapsulated fish oil using the one-step extraction method. Part 1: Measurement in the raw ingredient and in dry powdered foods. J. Am. Oil Chem. Soc..

[16] Sneddon E.J., Hardaway C.J., Sneddon J., Kiran B., Tate A.S., Tidwell S.L., Gray D.P., Douvris C. (2017). Determination of selected metals in rice and cereal by inductively coupled plasma-optical emission spectrometry (ICP-OES). Microchem. J..

[17] Chotphruethipong L., Sukketsiri W., Aluko R.E., Sae-Leaw T., Benjakul S. (2021). Effect of hydrolyzed collagen from defatted Asian sea bass (*Lates calcarifer*) skin on fibroblast proliferation, migration and antioxidant activities. J. Food Sci. Technol..

[18] Suttithumsatid W., Shah M.A., Bibi S., Panichayupakaranant P. (2022). α-Glucosidase inhibitory activity of cannabidiol, tetrahydrocannabinol and standardized cannabinoid extracts from *Cannabis sativa*. Curr. Res. Food Sci..

[19] Boonruamkaew P., Sukketsiri W., Panichayupakaranant P., Kaewnam W., Tanasawet S., Tipmanee V., Hutamekalin P., Chonpathompikunlert P. (2017). *Apium graveolens* extract influences mood and cognition in healthy mice. J. Nat. Med..

[20] Suttithumsatid W., Sukketsiri W., Panichayupakaranant P. (2023). Cannabinoids and standardized cannabis extracts inhibit migration, invasion, and induce apoptosis in MCF-7 cells through FAK/MAPK/Akt/NF-κB signaling. Toxicol. In Vitro.

[21] Kukusamude C., Sricharoen P., Limchoowong N., Kongsri S. (2021). Heavy metals and probabilistic risk assessment via rice consumption in Thailand. Food Chem..

[22] Dewan M.F., Ahiduzzaman M., Islam M.N., Shozib H.B. (2023). Potential benefits of bioactive compounds of traditional rice grown in South and Southeast Asia: A review. Rice Sci..

[23] Zhang Q.W., Lin L.G., Ye W.C. (2018). Techniques for extraction and isolation of natural products: A comprehensive review. Chin. Med..

[24] Alara O.R., Abdurahman N.H., Olalere O.A. (2020). Ethanolic extraction of flavonoids, phenolics and antioxidants from *Vernonia amygdalina* leaf using two-level factorial design. J. King Saud. Univ. Sci..

[25] Chaves J.O., de Souza M.C., da Silva L.C., Lachos-Perez D., Torres-Mayanga P.C., Machado A.F., Forster-Carneiro T., Vázquez-Espinosa M., González-de-Peredo A.V., Barbero G.F. (2020). Extraction of flavonoids from natural sources using modern techniques. Front. Chem..

[26] Shi L., Zhao W., Yang Z., Subbiah V., Suleria H.A.R. (2022). Extraction and characterization of phenolic compounds and their potential antioxidant activities. Environ. Sci. Pollut. Res..

[27] Jayaprakash G., Bains A., Chawla P., Fogarasi M., Fogarasi S. (2022). A narrative review on rice proteins: Current scenario and food industrial application. Polymers.

[28] Melgosa R., Marques M., Paiva A., Bernardo A., Fernández N., Sá-Nogueira I., Simões P. (2021). Subcritical water extraction and hydrolysis of Cod (*Gadus morhua*) frames to produce bioactive protein extracts. Foods.

[29] Sitanggang A.B., Joshua M., Munarko H., Kusnandar F., Budijanto S. (2021). Increased γ-aminobutyric acid content of germinated brown rice produced in membrane reactor. Food Technol. Biotechnol..

[30] Kalman D.S. (2014). Amino acid composition of an organic brown rice protein concentrate and isolate compared to soy and whey concentrates and isolates. Foods.

[31] Ribas F.B.T., Gasparetto H., Salau N.P.G. (2023). Sustainable extraction of rice bran oil: Assessing renewable solvents, kinetics, and thermodynamics. Chem. Eng. Res. Des..

[32] Ilias N.N., Mohd Rozalli N.H., Mohamad Kassim M.H. (2023). Characterizations of rice bran nanofibers produced by enzymatic treatment and their role in stabilizing oil-in-water pickering emulsions. Waste Biomass Valorization.

[33] Özgül-Yücel S., Proctor A. (2004). Rice bran FFA determination by diffuse reflectance IR spectroscopy. J. Am. Oil Chem. Soc..

[34] Prasad P., Savyasachi S., Reddy L.P.A., Sreedhar R.V. (2019). Physico-chemical characterization, profiling of total lipids and triacylglycerol molecular species of omega-3 fatty acid rich B. arvensis seed oil from India. J. Oleo Sci..

[35] Falcioni R., Moriwaki T., Gibin M.S., Vollmann A., Pattaro M.C., Giacomelli M.E., Sato F., Nanni M.R., Antunes W.C. (2022). Classification and prediction by pigment content in Lettuce (*Lactuca sativa* L.) varieties using machine learning and ATR-FTIR spectroscopy. Plants.

[36] Mohammed E.A., Abdalla I.G., Alfawaz M.A., Mohammed M.A., Al Maiman S.A., Osman M.A., Yagoub A.E.A., Hassan A.B. (2022). Effects of extraction solvents on the total phenolic content, total flavonoid content, and antioxidant activity in the aerial part of root vegetables. Agriculture.

[37] Kaur S., Ubeyitogullari A. (2023). Extraction of phenolic compounds from rice husk via ethanol-water-modified supercritical carbon dioxide. Heliyon.

[38] Wanyo P., Kaewseejan N., Meeso N., Siriamornpun S. (2016). Bioactive compounds and antioxidant properties of different solvent extracts derived from Thai rice by-products. Appl. Biol. Chem..

[39] Scarano A., Laddomada B., Blando F., De Santis S., Verna G., Chieppa M., Santino A. (2023). The chelating ability of plant polyphenols can affect iron homeostasis and gut microbiota. Antioxidants.

[40] Del-Toro-Sánchez C.L., Rodríguez-Félix F., Cinco-Moroyoqui F.J., Juárez J., Ruiz-Cruz S., Wong-Corral F.J., Borboa-Flores J., Castro-Enríquez D.D., Barreras-Urbina C.G., Tapia-Hernández J.A. (2021). Recovery of phytochemical from three safflower (*Carthamus tinctorius* L.) by-products: Antioxidant properties, protective effect of human erythrocytes and profile by UPLC-DAD-MS. J. Food Process Preserv..

[41] Chaurasiya N.D., Leon F., Muhammad I., Tekwani B.L. (2022). Natural products inhibitors of monoamine oxidases-potential new drug leads for neuroprotection, neurological disorders, and neuroblastoma. Molecules.

[42] Ademosun A.O., Oboh G. (2014). Comparison of the inhibition of monoamine oxidase and butyrylcholinesterase activities by infusions from green tea and some citrus peels. Int. J. Alzheimers Dis..

[43] Ganiyu O., Olunbamigbe O.O., Ogunsuyi O.B., Aro O.P., Oyeleye I.S., Ademosun A.O. (2024). Evaluating the nutrient composition and antioxidant properties of orange (*Citrus sinensis*) peels through Penicillium camemberti-based solid-substrate fermentation. Discov. Food.

[44] Moelands S.V., Lucassen P.L., Akkermans R.P., De Grauw W.J., Van de Laar F.A. (2018). Alpha-glucosidase inhibitors for prevention or delay of type 2 diabetes mellitus and its associated complications in people at increased risk of developing type 2 diabetes mellitus. Cochrane Database Syst. Rev..

[45] Chaachouay N., Zidane L. (2024). Plant-derived natural products: A source for drug discovery and development. Drugs Drug Candidates.

[46] Bhuyan P., Ganguly M., Baruah I., Borgohain G., Hazarika J., Sarma S. (2022). Alpha glucosidase inhibitory properties of a few bioactive compounds isolated from black rice bran: Combined in vitro and in silico evidence supporting the antidiabetic effect of black rice. RSC Adv..

[47] Sansenya S., Payaka A. (2022). Inhibitory potential of phenolic compounds of Thai colored rice (*Oryza sativa* L.) against α-glucosidase and α-amylase through in vitro and in silico studies. J. Sci. Food Agric..

[48] Laka K., Makgoo L., Mbita Z. (2022). Cholesterol-lowering phytochemicals: Targeting the mevalonate pathway for anticancer interventions. Front. Genet..

[49] Sukketsiri W., Daodee S., Parhira S., Malakul W., Tunsophon S., Sutthiwong N., Tanasawet S., Chonpathompikunlert P. (2023). Chemical characterization of *Passiflora edulis* extracts and their in vitro antioxidant, anti-inflammatory, anti-lipid activities, and ex-vivo vasodilation effect. J. King Saud. Univ. Sci..

[50] Heres A., Mora L., Toldrá F. (2021). Inhibition of 3-hydroxy-3-methyl-glutaryl-coenzyme A reductase enzyme by dipeptides identified in dry-cured ham. Food Prod. Process Nutr..

[51] Rizeq B., Gupta I., Ilesanmi J., AlSafran M., Rahman M.M., Ouhtit A. (2020). The power of phytochemicals combination in cancer chemoprevention. J. Cancer.

[52] Wei Z., Liu X., Cheng C., Yu W., Yi P. (2020). Metabolism of amino acids in cancer. Front. Cell Dev. Biol..

[53] Lieu E.L., Nguyen T., Rhyne S., Kim J. (2020). Amino acids in cancer. Exp. Mol. Med..

[54] Hardman W.E. (2002). Omega-3 fatty acids to augment cancer therapy. J. Nutr..

